# Abnormalities of Localized Connectivity in Obsessive-Compulsive Disorder: A Voxel-Wise Meta-Analysis

**DOI:** 10.3389/fnhum.2021.739175

**Published:** 2021-09-16

**Authors:** Xiuli Qing, Li Gu, Dehua Li

**Affiliations:** ^1^Department of Obstetrics, Key Laboratory of Birth Defects and Related Diseases of Women and Children in Ministry of Education, West China Second University Hospital, Sichuan University, Chengdu, China; ^2^Nursing Department, West China Second University Hospital, Sichuan University, Chengdu, China

**Keywords:** obsessive-compulsive disorder, resting-state functional magnetic resonance imaging, localized connectivity, regional homogeneity, meta-analysis, seed-based d mapping

## Abstract

**Background:** A large amount of resting-state functional magnetic resonance imaging (rs-fMRI) studies have revealed abnormalities of regional homogeneity (ReHo, an index of localized intraregional connectivity) in the obsessive-compulsive disorder (OCD) in the past few decades, However, the findings of these ReHo studies have remained inconsistent. Hence, we performed a meta-analysis to investigate the concurrence across ReHo studies for clarifying the most consistent localized connectivity underpinning this disorder.

**Methods:** A systematic review of online databases was conducted for whole-brain rs-fMRI studies comparing ReHo between OCD patients and healthy control subjects (HCS). Anisotropic effect size version of the seed-based d mapping, a voxel-wise meta-analytic approach, was adopted to explore regions of abnormal ReHo alterations in OCD patients relative to HCS. Additionally, meta-regression analyses were conducted to explore the potential effects of clinical features on the reported ReHo abnormalities.

**Results:** Ten datasets comprising 359 OCD patients and 361 HCS were included. Compared with HCS, patients with OCD showed higher ReHo in the bilateral inferior frontal gyri and orbitofrontal cortex (OFC). Meanwhile, lower ReHo was identified in the supplementary motor area (SMA) and bilateral cerebellum in OCD patients. Meta-regression analysis demonstrated that the ReHo in the OFC was negatively correlated with illness duration in OCD patients.

**Conclusions:** Our meta-analysis gave a quantitative overview of ReHo findings in OCD and demonstrated that the most consistent localized connectivity abnormalities in individuals with OCD are in the prefrontal cortex. Meanwhile, our findings provided evidence that the hypo-activation of SMA and cerebellum might be associated with the pathophysiology of OCD.

## Introduction

Obsessive-compulsive disorder (OCD), a common mental illness characterized persistent intrusive thoughts (obsessions) and/or ritualized repetitive behaviors (compulsions) (Stein et al., 2019), has a lifetime prevalence rate of 2 to 3% (Ruscio et al., 2010). OCD usually has an onset in childhood and turns into a chronic course (Ruscio et al., 2010). Despite its high disability rate and the resultant social burden, the neuropathology of OCD is still not fully understood. Thus, identifying the neural correlates of OCD is of paramount significance to elevate the diagnostic specificity and improve the treatment efficacy of this disorder.

The development of multimodal magnetic resonance imaging (MRI) techniques and neuroimage analytical approaches have greatly advanced our understanding of the neurobiological substrates regarding OCD in the past few decades (Dougherty et al., 2018). Previous structural MRI meta- and meta- analytical publications have indicated the key role of the cortico-striato-thalamo-cortical (CSTC) network in the pathophysiology of OCD (Radua and Mataix-Cols, 2009; Rotge et al., 2010; de Wit et al., 2014; Fouche et al., 2017; Hu et al., 2017). Meanwhile, it is reported that multiple phenotypic subtypes of OCD might have different structural neural substrates (Dougherty et al., 2018). For example, Hirose et al. found a negative association between washing symptom dimension score and the right thalamic gray matter as well as a significant negative correlation between hoarding symptom dimension score and the left angular white matter in OCD patients (Hirose et al., 2017). In terms of the functional MRI (fMRI) researches in OCD, the results appear to be highly heterogeneous. For example, patients with OCD showed abnormal activation of mesolimbic and ventral striatal circuitry during reward-based spatial learning (Marsh et al., 2015). One experiment testing the error monitoring function revealed hyperactivation of the right amygdala and the subgenual anterior cingulate cortex in OCD patients compared with healthy control subjects (HCS) (Grutzmann et al., 2016). Another fMRI study examining decision making function found that OCD patients showed hypo-activation in the ventromedial orbitofrontal cortex (Norman et al., 2018). The discrepancies between these fMRI studies might be attributed to sample size, clinical heterogeneity (such as medication strategies and comorbidity profiles) and experimental paradigm, which dramatically affected the fMRI findings.

Rather than traditional task-based fMRI, the resting-state fMRI (rs-fMRI) is a commonly used neuroimaging approach to explore the brain function alterations in normal and disease states without performing any task (Biswal, 2012). The amplitude low-frequency Puctuation (ALFF) is a commonly used rs-fMRI parameter that could provide information of regional activation of brain (Fox and Raichle, 2007) while an improved measure named fractional amplitude of low-frequency fluctuation (fALFF) has been put forward as a normalized version of ALFF (Zou et al., 2008). Previous investigations have demonstrated alterations of (f)ALFF in a range of brain regions including the classical CSTC circuits and some newly found brain areas such as the parietal lobe, temporal lobe and the cerebellum (Hou et al., 2012; Fan et al., 2017; Gimenez et al., 2017; Qiu et al., 2017). Besides the (f)ALFF, functional connectivity (FC), a valid rs-fMRI index reflecting the level of integration of local activity across brain regions (Buckner et al., 2013), has been widely adopted to investigate the neural pathogenesis of OCD (Gursel et al., 2018). Previous FC studies have identified that, besides the classical CSTC circuitry, the between-network hypoconnectivity of triple-network (salience, frontoparietal and default-mode networks) might also get involved in the psychopathology in OCD (Gursel et al., 2018). Though explorations of network-level neural function abnormalities in OCD have achieved remarkable progress, the local neural dysfunction of this disorder received less attention.

Regional Homogeneity (ReHo), a rs-fMRI parameter characterizing the local synchronization of spontaneous blood oxygen level-dependent signal fluctuation among neighboring voxels within a given cluster, offered new chance to investigate the localized connectivity disruptions in patients without a priori constraints (Zang et al., 2004). A large amount of rs-fMRI studies have revealed abnormalities of ReHo in OCD, However, the findings of these ReHo studies have remained inconsistent and controversial. For example, one study reported that OCD patients exhibited higher ReHo in the right cerebellum (Ping et al., 2013) while another study identified lower ReHo in the bilateral cerebellum of OCD patients (Hu et al., 2019). Thus, it was necessary to perform a quantitative overview of ReHo findings in OCD.

To our knowledge, Hao et al. published a meta-analysis concerning ReHo alterations in OCD via seed-based d mapping (SDM) approach (Hao et al., 2019). Nevertheless, there were two major shortcomings in their study. First, according to SDM designers' suggestion, the minimum of 10 studies was recommended for SDM meta-analyses (Carlisi et al., 2017; Muller et al., 2018). However, only eight datasets were included in their meta-analysis (Hao et al., 2019). Second, Hao et al. did not to evaluate the association between the clinical variables and ReHo alterations because the included studies were too few (less than 9 studies) to perform meta-regression analysis (Radua and Mataix-Cols, 2009). Therefore, we conducted an updated voxel wise meta-analysis to identify the most robust ReHo abnormalities in OCD patients compared with the controls using the Anisotropic effect size version of the seed-based d mapping (AES-SDM). This new version of SDM method has several advantages such as: (i) avoiding any voxel appearing significant in opposite directions; (ii) reconstructing both positive and negative differences in the same signed differential map; (iii) combining the reported peak coordinates with statistical parametric maps. Additionally, we performed meta-regression to explore the potential effects of clinical features on reported ReHo alterations.

## Materials and Methods

### Data Source

Systematic searches of the online database including PubMed, EMBASE and Web of Science (from January 2000 to December 2020) were conducted. The keyword searches were performed using the following terms: (“obsessive-compulsive disorder” or “OCD”) plus (“resting-state functional magnetic resonance imaging” or “rs-fMRI”) or (“regional homogeneity” or “ ReHo”) or (“localized connectivity”). We also screened the reference lists of relevant articles in order to obtain additional literature.

### Studies Selection and Data Extraction

A study was considered for inclusion if it (i) was a research paper and published in English; (ii) reported ReHo comparison between patients with OCD and HCS; (iii) provided 3-dimensional coordinates of ReHo abnormalities in stereotactic space at the whole-brain level; (iv) adopted significance thresholds for data that were corrected for multiple comparisons. In some cases, we obtained additional details which were essential for the meta-analysis by contacting the corresponding authors. Exclusion criteria were: (i) the article type of the study is not original investigation; (ii) the peak coordinates of the ReHo alterations could not be retrieved; (iii) the study was based on region of interest (ROI) analytical approach; (iv) the data overlapped with those of another publication. We performed the current meta-analysis based on the guidelines of Preferred Reporting Items for Systematic Reviews and Meta-Analysis (PRISMA) (Radua, 2021). The coordinates regarding the ReHo changes between OCD patients and HCS in each included study were independently extracted by two investigators. Meanwhile, clinical features (including the sample size, age, gender, illness duration, symptom severity and medication status) and methodological issues (such as the MRI scanner, analytical software, smoothing kernel, number of foci and the threshold for multiple comparison correction) were extracted. If agreement was not obtained, then another author mediated.

### Voxel-Wise Meta-Analysis

Using the AES-SDM software, we conducted the voxel-wise meta-analysis to explore the most robust ReHo abnormalities in patients with OCD compared with HCS based on the selected studies. Meanwhile, we performed a whole-brain jackknife sensitivity analyses to evaluate the reliability of the main effect. Afterwards, we conducted subgroup meta-analysis of unmedicated OCD patients and the subgroup meta-analysis regarding the threshold for correction was also performed. Subsequently, between-study variance was analyzed in order to assess significant heterogeneity of ReHo abnormalities. The kernel size and thresholds for the main effect and heterogeneity analysis were set as follows: full-width at half-maximum = 20 mm; anisotropy = 1.0, voxel *P* = 0.005, peak height threshold = 1, cluster extent = 100 voxels. We also performed Egger's test for the evaluation of publication bias. Finally, the meta-regression analyses were performed to examine the potential effects of clinical variables (such as symptom severity and illness duration) on the reported ReHo changes. It should be noted that a more conservative threshold (*P* < 0.0005) was used for meta-regression analysis in order to achieve the optimal balance of sensitivity and specificity as suggested by previous publication (Wise et al., 2016). All the analyses are performed according to the AES-SDM tutorial (http://www.sdmproject.com/software/Tutorial.pdf).

## Results

### Included Studies and Sample Characteristics

**Figure 1:**
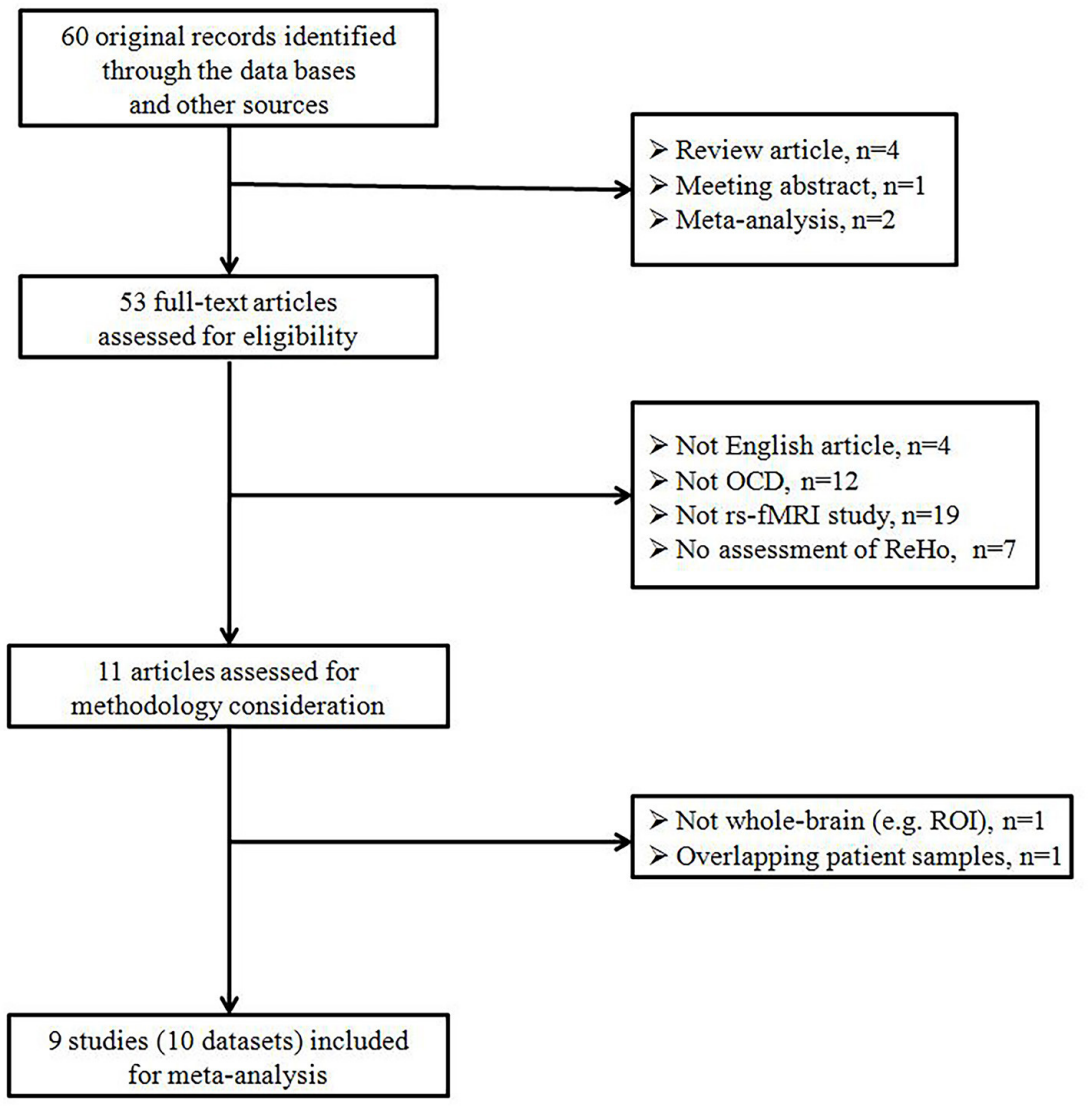
Flow diagram regarding the identification and attrition of studies. OCD, obsessive-compulsive disorder; ReHo, regional homogeneity; ROI, region of interest; rs-fMRI, resting-state functional magnetic resonance imaging.

Our search strategy identified a total of 60 studies. Of these, 11 ReHo studies were chosen for further consideration after primary screening. Among the 11 ReHo investigations, One study adopted an ROI analytical method instead of a whole-brain approach (Chen et al., 2016c). Another study recruited samples that were overlapped with previous publication (Chen et al., 2016b). Therefore, these two studies were excluded from the current meta-analysis. Ultimately, 9 original investigations (Yang et al., 2010, 2015, 2019; Ping et al., 2013; Chen et al., 2016a; Niu et al., 2017; Bu et al., 2019; Hu et al., 2019; Xia et al., 2020) met the inclusion criteria (see Figure 1 for details). No additional articles were found in the reference lists of the included studies. One investigation included two different subgroups of OCD patients (autogenous-type OCD and reactive-type OCD, two different types of obsessions in OCD proposed by Lee and Kwon, 2003; Xia et al., 2020). We treated this investigation as two unique datasets, with each patient subgroup selected independently in the current meta-analysis. Therefore, a total of 10 datasets comprising 359 OCD patients and 361 HCS were included in our meta-analysis, along with 80 coordinates extracted from these 10 datasets. There was no significant difference between the two groups in terms of age and sex. The mean age was 27.35 years in the OCD patient group vs. 26.56 years in the HCS group while there were 142 (39.6%) female OCD patients vs. 149 (41.2%) female HCS. The demographic details from all recruited studies were well-described in the Table 1 while the technical details of the included studies were available in the Table 2.

**Table 1:** Demographic and clinical characteristics of ReHo studies on OCD in the current meta-analysis.

	**Sociodemographic characteristics**						**Clinical characteristics: OCD participants only**				
**Study**	**No. of subjects**		**Mean age (yrs)**		**Female (%)**		**Mean illness duration (yrs)**	**Mean Y-BOCS score**	**Mean HAMA score**	**Mean HAMD score**	**Medication status (%)**
	**OCD**	**HCS**	**OCD**	**HCS**	**OCD**	**HCS**					
Yang et al., 2010	22	22	31.18	30.86	63.6	63.6	3.88	32.27	8.5	6.36	Drug-naïve
Ping et al., 2013	20	20	27.1	27.6	20	20	7.34	23.5	12.9	11.2	0.7
Yang et al., 2015	22	22	30.95	29.52	45.5	45.5	8.22	24.43	11.81	8.52	Drug-free
Chen et al., 2016a	30	30	26.23	28.17	20	23.3	5.54	23.77	12.8	10.8	0.67
Niu et al., 2017	26	25	24.19	22.68	30.8	52	5.49	22.92	14.35	15.58	Drug-naïve
Bu et al., 2019	54	54	30.41	28.39	37.1	37.1	8.15	20.72	9.24	8.19	Drug-free
Hu et al., 2019	88	88	29.16	27.88	36.4	36.4	7.32	21.47	8.78	8.74	Drug-free
Yang et al., 2019	15	30	28.77	28.23	60	33.3	7.15	25	6.54	7.23	Drug-free
Xia et al., 2020^#^	40	70	22.48	20.93	45	55.7	4.08	21.63	NA	NA	Drug-free
Xia et al., 2020^*^	42	70	22.76	20.93	50	55.7	4.33	22.6	NA	NA	Drug-free

*HAMA, Hamilton Anxiety Rating Scale; HAMD, Hamilton Depression Rating Scale; HCS, healthy control subjects; OCD, obsessive-compulsive disorder; ReHo, regional homogeneity; Y-BOCS, Yale-Brown Obsessive Compulsive Scale.*

# *Subgroup of autogenous-type OCD patients.*

* *Subgroup of reactive-type OCD patients*.

**Table 2:** Technical details of ReHo studies on OCD in the current meta-analysis.

**Study**	**MRI Scanner**	**Software**	**Smoothing (FWHM)**	**Coordinate System**	**Foci**	***p*-value (correction)**
Yang et al., 2010	1.5T (GE)	SPM8	10 mm	MNI	3	*p* < 0.05 (AlphaSim corrected)
Ping et al., 2013	3.0T (Siemens)	SPM5	4 mm	MNI	20	*p* < 0.05 (AlphaSim corrected)
Yang et al., 2015	3.0T (Siemens)	SPM5	4 mm	MNI	11	*p* < 0.05 (AlphaSim corrected)
Chen et al., 2016a	3.0T (GE)	SPM8	4 mm	MNI	10	*p* < 0.05 (AlphaSim corrected)
Niu et al., 2017	3.0T (GE)	SPM8	4 mm	MNI	5	*p* < 0.005 (AlphaSim corrected)
Bu et al., 2019	3.0T (GE)	SPM8	8 mm	MNI	0	*p* < 0.05 (FDR corrected)
Hu et al., 2019	3.0T (GE)	SPM8	8 mm	MNI	16	*p* < 0.05 (FWE corrected)
Yang et al., 2019	3.0T (Siemens)	DPABI-V	4 mm	MNI	7	*p* < 0.05 (GRF corrected)
Xia et al., 2020^#^	3.0T (Siemens)	SPM12	6 mm	MNI	5	*p* < 0.05 (FDR corrected)
Xia et al., 2020^*^	3.0T (Siemens)	SPM12	6 mm	MNI	3	*p* < 0.05 (FDR corrected)

*DPABI, data processing and analysis for brain imaging; FDR, false discovery rate; FWE, family wise error; FWHM, full width at half maximum; GRF, Gaussian random field; MNI, Montreal Neurological Institute; OCD, obsessive-compulsive disorder; ReHo, regional homogeneity; SPM, statistical parametric mapping; T, Tesla.*

# *Subgroup of autogenous-type OCD patients.*

* *Subgroup of reactive-type OCD patients*.

### Regional ReHo Differences Between Patients With OCD and HCS

**Figure 2:**
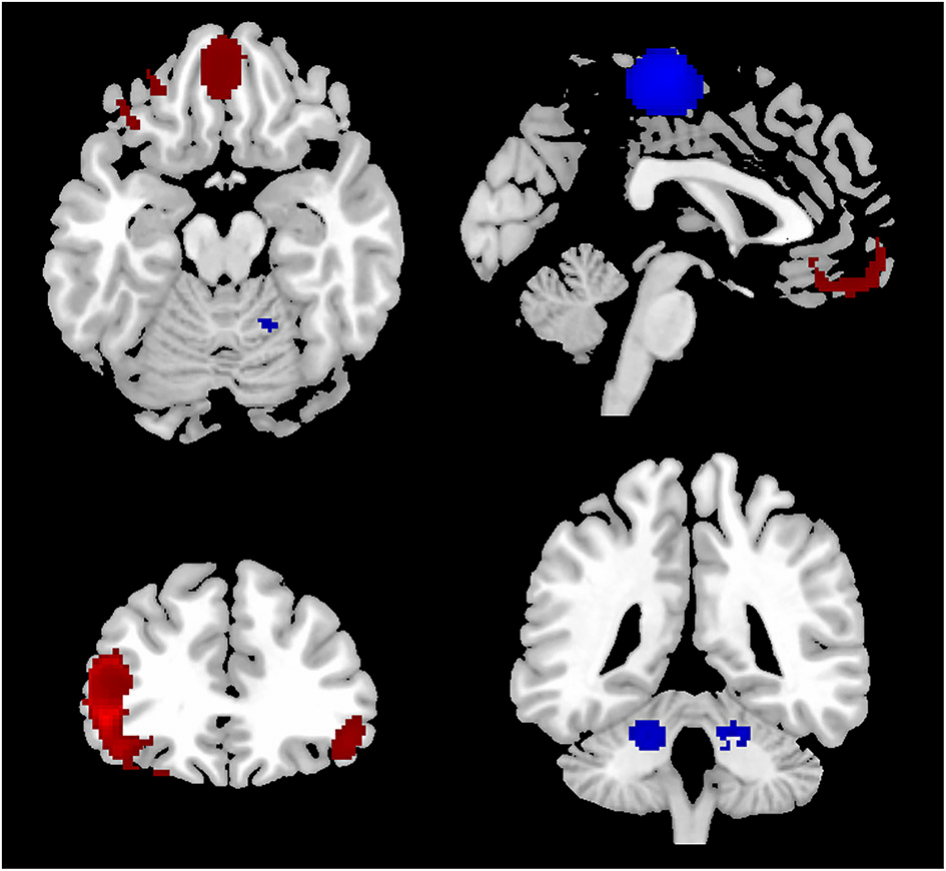
Results of the meta-analysis of whole-brain ReHo studies in patients with OCD. Regions of ReHo alterations in patients with OCD relative to HCS were shown on the three-dimensional T1-weighted template images from software (MRIcroN). Higher ReHo was shown in red color while lower ReHo was displayed in blue color. HCS, healthy control subjects; OCD, obsessive-compulsive disorder; ReHo, regional homogeneity.

Compared with HCS, patients with OCD showed higher ReHo in the bilateral inferior frontal gyri (IFG) and orbitofrontal cortex (OFC). Meanwhile, lower ReHo was identified in the supplementary motor area (SMA) and bilateral cerebellum in OCD patients (see Figure 2 and Table 3 for details). All aforementioned clusters did not reveal significant statistical heterogeneity between studies (*p* > 0.005). Additionally, none of the clusters showed significant publication bias in the Egger's test (*p* > 0.05).

**Table 3:** Statistical concurrence observed across ReHo studies on OCD.

**Region**	**Local maximum**					**Cluster**		**Jackknife sensitivity analysis (combination of studies detecting the differences)**
	**MNI Coordinates**			**SDM-Z**	***P***	**Number of voxels**	**Breakdown (number of voxel)**	
**Higher ReHo (OCD** **>** **HCS)**								
Left inferior frontal gyrus	−48	34	0	3.203	~0	2023	Left inferior frontal gyrus (1663)	10 out of 10
							Left middle frontal gyrus (202)	
							Left insula (158)	
Right inferior frontal gyrus	48	36	−10	1.942	0.000668841	408	Right inferior frontal gyrus (370)	8 out of 10
							Right middle frontal gyrus (38)	
Left orbitofrontal gyrus	−10	50	−16	1.792	0.001478572	587	Left orbitfrontal gyrus (339)	8 out of 10
							Right orbitfrontal gyrus (248)	
**Lower ReHo (OCD** **<** **HCS)**								
Right supplementary motor area	6	−20	66	−1.996	0.000082057	1443	Left paracentral lobule (327)	10 out of 10
							Left supplementary motor area (129)	
							Right paracentral lobule (363)	
							Right precentral gyrus (96)	
							Right supplementary motor area (528)	
Left cerebellum	−14	−52	−24	−1.605	0.001129702	269	Left cerebellum (269)	9 out of 10
Right cerebellum	20	−58	−26	−1.604	0.001129702	160	Left cerebellum (160)	9 out of 10

*HCS, healthy control subjects; MNI, Montreal Neurological Institute; OCD, obsessive-compulsive disorder; ReHo, regional homogeneity; SDM, seed-based d mapping*.

### Subgroup Meta-Analyses

The subgroup meta-analyses showed that the main findings above remained highly reproducible when only the 8 ummedicated OCD datasets or only the 9 datasets using the threshold of 0.05 for multiple comparison corrections were analyzed (Table 4). Unfortunately, we failed to perform the subgroup meta-analyses regarding other clinical subtypes or imaging methodologies because there were not enough primary datasets.

**Table 4:** Sensitivity analyses of clusters with altered ReHo between OCD patients and controls from 9 included studies (10 datasets) in the current meta-analysis.

**Analysis**	**Left IFG**	**Right IFG**	**Left OFG**	**Right SMA**	**Left cerebellum**	**Right cerebellum**
**Jackknife sensitivity analysis (discarded study)**						
Yang et al., 2010	Y	Y	Y	Y	Y	Y
Ping et al., 2013	Y	Y	Y	Y	Y	Y
Yang et al., 2015	Y	N	Y	Y	Y	Y
Chen et al., 2016a	Y	N	Y	Y	Y	Y
Niu et al., 2017	Y	Y	Y	Y	Y	Y
Bu et al., 2019	Y	Y	Y	Y	Y	Y
Hu et al., 2019	Y	Y	Y	Y	N	N
Yang et al., 2019	Y	Y	Y	Y	Y	Y
Xia et al., 2020^#^	Y	Y	N	Y	Y	Y
Xia et al., 2020^*^	Y	Y	N	Y	Y	Y
**Subgroup analysis**						
Studies including unmedicated OCD patients (*N* = 8)	Y	N	Y	Y	Y	Y
Studies corrected using threshold of 0.05 (*N* = 9)	Y	Y	Y	Y	Y	Y

*IFG, inferior frontal gyrus; N, no; OCD, obsessive-compulsive disorder; OFG, orbitofrontal gyrus; ReHo, regional homogeneity;. SMA, supplementary motor area; Y, yes.*

# *Subgroup of autogenous-type OCD patients.*

* *Subgroup of reactive-type OCD patients*.

### Sensitivity Analyses

As displayed in the Table 3, the whole brain jackknife sensitivity analyses indicated that higher ReHo in the left IFG and lower ReHo in the SMA were highly replicable, because these two findings were consistent throughout all the 10 combinations of 9 datasets. The lower ReHo in the bilateral cerebellum failed to emerge in one of the study combinations while the higher ReHo in the right IFG and OFC failed to emerge in two of the study combinations. The detailed results of the whole brain jackknife sensitivity analyses were shown in the Table 4.

### Meta-Regression Analysis

**Figure 3:**
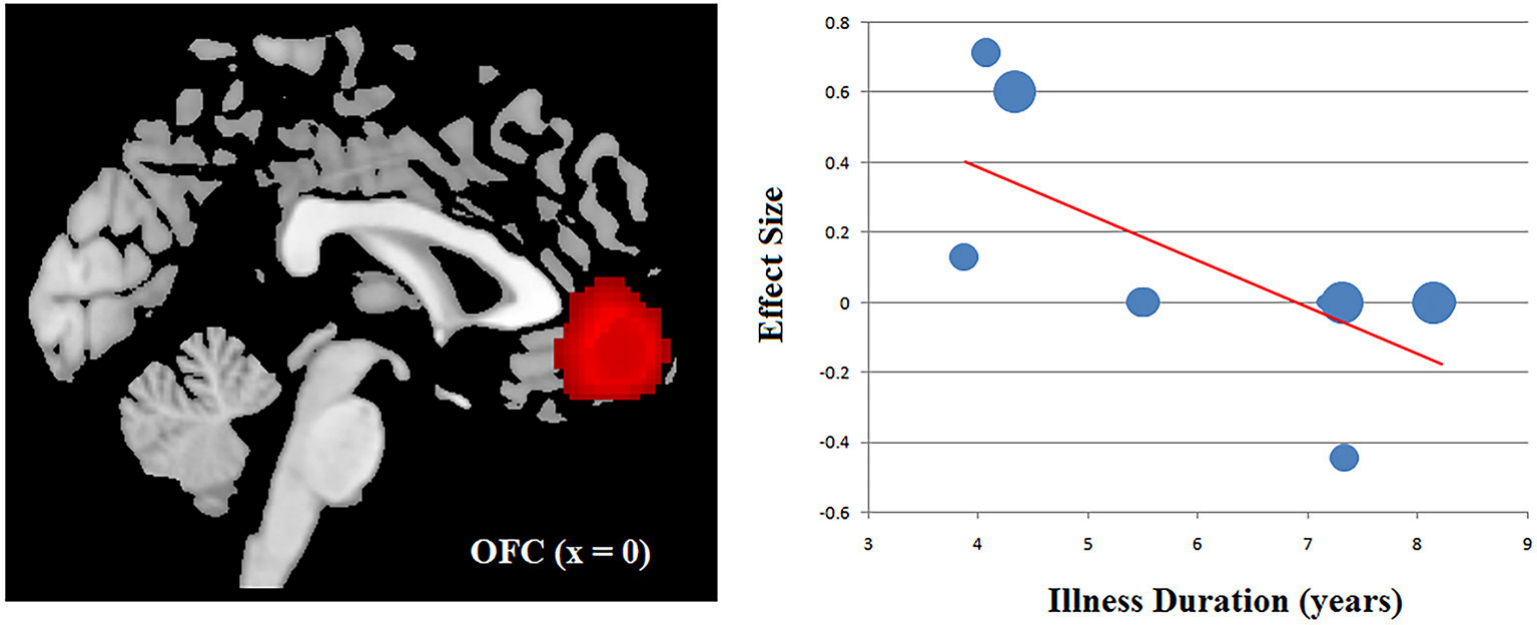
Results of meta-regression analysis illustrating a negative association between the ReHo in the OFC and the illness duration in patients with OCD. The effects sizes were extracted to create the plots in the graph and each study is represented as a dot, with dot size reflecting sample size: large dots indicate samples with over 40 patients; medium dots, samples with 20–40 patients; and small dots, samples with under 20 patients. OCD, obsessive-compulsive disorder; OFC, orbitofrontal cortex; ReHo, regional homogeneity.

The clinical Information of the patients with OCD including the age, gender, symptom severity and illness duration was available for all the 10 datasets. Using a stringent threshold of *P* < 0.0005 to minimize spurious findings, our meta regression revealed that samples with longer illness duration of OCD patients had more decreased ReHo in the OFC, which had been found as anomalous in the main effect. That is, the illness duration was negatively associated with the ReHo in the OFC (x = 0, y = 46, z = −2; SDM-Z = −3.304, *P* = 0.000005677; 428 voxels) (Figure 3). Other relevant clinical variables were not correlated, at least linearly, with OCD-related ReHo alterations.

## Discussion

The current study integrated rs-fMRI publications for a meta-analysis of ReHo differences between OCD patients and HCS. Using AES-SDM approach, our meta-analysis identified that patients with OCD showed higher ReHo in the bilateral IFG and OFC. Meanwhile, lower ReHo was identified in the SMA and bilateral cerebellum in OCD patients. These findings remained stable when jackknife sensitivity analyses were performed, which suggested that the results of our meta-analysis were robust and reliable.

In line with the classical CSTC model of OCD, we identified higher ReHo in the bilateral IFG and OFC in OCD patients relative to the HCS. The prefrontal dysfunction is widely considered to be implicated in the psychopathology of OCD (Pauls et al., 2014). Localized connectivity dysfunction in bilateral IFG might be associated with impairments of cognitive control, which had been consistently reported in OCD patients (Shin et al., 2014). Previous multicenter mega-analytical publication has demonstrated smaller gray matter volume in bilateral IFG in OCD patients while the current study revealed higher ReHo in the bilateral IFG (de Wit et al., 2014). We speculated that the hyper-activation of bilateral IFG is a compensatory response to the gray matter structural deficits of IFG. It is reported that the OFC plays an essential role in reward processing (Milad and Rauch, 2012). Recent meta-analysis has demonstrated lower fractional anisotropy in the left orbitofrontal white matter of OCD patients, which was negatively and independently associated with symptom severity and illness duration in patients with OCD (Hu et al., 2020). One animal experiment indicated that giving repeated stimulation to the OFC of the mice could lead to persistent OCD-like behaviors (Ahmari et al., 2013). Grover et al. found that high-frequency neuromodulation of OFC could improve obsessive-compulsive behavior (Grover et al., 2021). In the current study, higher ReHo in the OFC may be related the behavioral deficits of OCD patients since OCD patients perform poorly on tasks that require adjusting responses based on changing reward feedback (Marsh et al., 2015). Additionally, our meta-regression analysis showed a negative correlation between the illness duration and the ReHo in the OFC. Previous study demonstrated a negative association between disease duration and ReHo value in the bilateral OFC in OCD patients at the whole-brain level (Niu et al., 2017). Yun et al. performed a multicenter study and found the centrality of orbito-frontal cortical surface areas was negatively correlated with OCD illness duration (Yun et al., 2020). Based on the evidence above, we proposed that the OFC might be related to the illness chronicity in OCD. However, this meta-regression finding should be interpreted with caution since two datasets (Ping et al., 2013; Chen et al., 2016a) in the current meta-analysis included OCD patients who were on stable doses of serotonin reuptake inhibitors at the time of the MRI scanning. Beucke et al. reported that antidepressant medication might affect the neural function within the CSTC circuits in OCD (Beucke et al., 2013). Therefore, further studies would be warranted to clarify our meta-regression finding.

It should be noted that prior meta-analysis reported decreased ReHo in the left caudate nucleus (Hao et al., 2019) while our meta analysis identified no ReHo alterations in the striatum. One possible reason accounting for the inconsistency is the differences of included datasets. A larger number of datasets was included in the current meta analysis (*N* = 10) than in the previous publication (*N* = 8). As suggested by Radua and his colleagues, the minimum of 10 studies was essential for the reliability of performing the SDM meta-analysis (Carlisi et al., 2017; Muller et al., 2018). Therefore, we confirmed the validity of the current meta-analysis.

The SMA is considered to be implicated in movement initiation and inhibition, response selection, and motor planning (Bonini et al., 2014). A task-based MRI study demonstrated that OCD patients and their siblings showed greater activity in the left SMA during successful inhibition paradigm relative to HCS, indicating that the SMA hyperactivity is a neurocognitive endophenotype of OCD (de Wit et al., 2012). Another study found that increased correlation of the error-related negativity in the event-related potential and activation of SMA might indicate stronger recruitment of proactive control in OCD (Grutzmann et al., 2016). Our meta-analysis revealed lower ReHo in the SMA, suggesting that hypo-activation of the SMA might be involved the pathophysiology of OCD.

Another interesting finding is that we identified lower ReHo in the bilateral cerebellum in OCD patients. Besides the traditional role of motor control, researches have proved that the cerebellum is involved in cognitive control (Buckner, 2013) and information processing (Ramnani, 2006). In fact, the cerebellum offers output to the cerebral cortex and tunes sensory input for facilitating behavioral adjustment in response to feedback (Gao et al., 2018). Sha et al. reported greater somatomotor-cerebellar connectivity in OCD patients and highlighted somatomotor-cerebellar circuits as potential targets for novel treatments in OCD (Sha et al., 2020). One study demonstrated decreased dynamic amplitude of low-frequency fluctuation (dALFF) of cerebellum in drug-naive OCD patients using the sliding-window approach (Liu et al., 2021). Meanwhile, another rs-fMRI study identified decreased cerebellar-cerebral functional connectivity in executive control and emotion processing networks in OCD patients (Xu et al., 2019). Taken collectively, our meta analysis emphasized the role of cerebellum in the pathogenesis of OCD.

In terms of the significance of ReHo alterations, previous investigations suggested that the index of ReHo could contribute the blood oxygenation level dependent (BOLD) fluctuations at the baseline (Anderson et al., 2014). An elevation of prefrontal ReHo might suggest an pronounced participation of this brain region in the neurophysiological functions such as the rumination (Dar and Iqbal, 2015) while a reduction of ReHo usually occurs alongside an increase in distributed connectivity during late neurodevelopment (Fair et al., 2007; Supekar et al., 2009).

Several limitations of the current meta-analysis should be addressed. First, our meta-analysis was performed on the basis of stereotactic coordinates extracted from each included dataset instead of raw brain maps (Radua et al., 2012), which might result in less accurate findings. Second, as the number of datasets included in our meta analysis was small, we failed to perform subgroup meta-analyses. Third, the potential effects of drug treatment could not be fully ruled out since a majority of studies employing OCD patients who were on drug treatment. Future ReHo studies recruiting unmedicated OCD patients are still needed to verify the reproducibility of the findings in the current meta analysis. Forth, it should be pointed out that all the included studies were conducted in China, which limited the generalizability of the our findings to other populations. Finally, the meta-regression results should be regarded as preliminary finding rather than conclusive evidence because the number of eligible studies for meta-regression analysis is limited.

In summary, the current meta-analysis presented a quantitative overview of ReHo findings in OCD and demonstrated that the most consistent localized connectivity abnormalities in individuals with OCD are in the prefrontal cortex. Additionally, our findings provided evidence that the hypo-activation of SMA and cerebellum might be associated with the pathophysiology of OCD, which might give additional explanation to the well known CSTC model of OCD.

## Data Availability Statement

The original contributions presented in the study are included in the article/Supplementary Material, further inquiries can be directed to the corresponding author/s.

## Author Contributions

LG and DL designed the study. XQ and LG acquired the data and wrote the article, which DL reviewed. XQ, LG, and DL analyzed the data. All authors approved the final version for publication.

## Conflict of Interest

The authors declare that the research was conducted in the absence of any commercial or financial relationships that could be construed as a potential conflict of interest. The reviewer XY declared a shared affiliation, with no collaboration, with the authors to the handling editor at the time of the review.

## Publisher's Note

All claims expressed in this article are solely those of the authors and do not necessarily represent those of their affiliated organizations, or those of the publisher, the editors and the reviewers. Any product that may be evaluated in this article, or claim that may be made by its manufacturer, is not guaranteed or endorsed by the publisher.
